# The transition from university to work: what happens to mental health? A longitudinal study

**DOI:** 10.1186/s40359-019-0340-x

**Published:** 2019-10-16

**Authors:** Amy Østertun Geirdal, Per Nerdrum, Tore Bonsaksen

**Affiliations:** 1 Faculty of Social Sciences, Department of Social Work, Child Welfare and Social Policy, Oslo Metropolitan University, PB 4 St. Olavs plass, N-0130 Oslo, Norway; 2 Centre for Senior Citizen Staff, Oslo Metropolitan University, PB 4 St. Olavs plass, N-0130 Oslo, Norway; 3 Faculty of Health Sciences, Department of Occupational Therapy, Prosthetics and Orthotics, Oslo Metropolitan University, PB 4 St. Olavs plass, N-0130 Oslo, Norway

**Keywords:** Professions, Psychological distress, Psychosocial work environment

## Abstract

**Background:**

When enrolled in university or college, students receive varying degrees of training in managing practical situations in the workplace. However, after graduation, the young professionals meet their responsibilities at work. The experience of the transition between education and work may connote a feeling of professional uncertainty and lack of coping, both of which are important factors related to young professionals’ mental health. The gap between the two areas of knowledge is frequently described as ‘practice shock’. Very few studies of mental health among students and young professional workers have used longitudinal designs. In the present study, we conducted a longitudinal investigation of change and stability in the levels of psychological distress among healthcare professionals, teachers, and social workers from the end of their study programs until 3 years into their subsequent professional lives. We also assessed the extent to which psychological distress at the end of the study program, sociodemographic characteristics, coping with the professional role, the psychosocial workplace environment, and experience of overall quality of life can predict psychological distress 3 years into their professional lives.

**Methods:**

Psychological distress was measured using the General Health Questionnaire 12 (GHQ-12). A total of 773 students/young professionals participated at both the end of their study programs and 3 years into their professional lives. Group differences were examined by the chi-squared test, independent samples *t*-test, and one-way analysis of variance. McNemar’s test were applied to identify changes in the proportion of cases at the two time points. Linear and logistic regressions were employed to identify factors associated with GHQ-12 Likert scores and GHQ-12 case scores, respectively.

**Results:**

Psychological distress was significantly reduced at 3 years for health professionals. Among the social workers and teachers, the change in psychological distress was not significant during the same period. Higher current quality of life contributed to lower psychological distress.

**Conclusions:**

Our findings support assumptions about higher levels of mental health problems as students, with mental health improving as health professionals and social workers move into professional work.

## Background

A person’s time living as a student comprises some of the most important activities in their life. We study to acquire new knowledge, enter new roles, find close friends, and establish intimate relationships. Most of all, as students, we prepare for life as a professional worker. During the first years in work, we try to integrate and practice the skills in which we were trained during our education. From an educational perspective, this change in context may create a gap between the theoretical knowledge obtained at the university and the practical knowledge expected from young professionals in the workplace. Experiencing this transition may connote a feeling of professional uncertainty and lack of coping, both of which are important factors related to young professionals’ mental health. This gap between the two areas of knowledge is frequently described as ‘practice shock’ [1–3] or ‘transfer shock’ [4].

The World Health Organization (WHO) defines mental health as “a state of well-being in which every individual realizes his or her own potential, can cope with the normal stresses of life, can work productively and fruitfully and is able to make a contribution to her or his community” [5]. According to the WHO, positive mental health is conceptualized as positive emotions, such as feelings of happiness, and personal factors, including psychological resources such as self-esteem and mastery [6]. Ill mental health has a negative impact on an individual’s quality of life and ability to function adequately [5]. These three definitions describe mental health in students, as well as among professional workers, and are operationalized in several instruments with high reliability and validity, including the Beck Depression Inventory (BDI), General Health Questionnaire 12 (GHQ-12), and Hopkins Symptom Checklist 90 (HSCL-90) [7–9].

Many studies of the mental health of students exist, and at least an equivalent number of studies have been concerned with mental health among persons in professional work. Almost all of these studies of mental health among students and professional workers have used a cross-sectional design.

Most studies of students claim that there is a clear tendency for higher education to be associated with deteriorations in students` mental health. The large American Freshman study [10] presented data from 153,015 students, including their self-rated emotional health. From 2009 to 2014, the proportion of students who “frequently” felt depressed increased from 6.1 to 9.5%. The annual student health report from the American College Health Association (ACHA) [11] reported similar findings. From 2009 (30.7%) to 2015 (34.5%), approximately 90,000 students reported that they had “felt so depressed that it was difficult to function” at any time during the last year. Since 2015, roughly 45,000 Canadian students have participated in ACHA monitoring. Among the Canadian students, an even higher proportion (44%) reported the same level of depression at any time during the last year. Even if the methods of measurement were more or less the same, none of the cited studies have reported longitudinal data on the students’ development over time.

Qualitative studies on students’ mental health in the UK have found a similar tendency, as presented in a report from the Royal College of Psychiatrists [12]. They stated that students in higher education exhibit increased symptoms of mental illness. The UK reports of increased mental illness among students may be a consequence of narrowing the treatment services on campus [13]. Rickinson and Turner [14] stated that in trying to understand this increase, it is important to bear in mind that “people are integral to the system in which they function”. The UK studies have been criticized for their lack of hard data [13].

The 2010 and 2014 Norwegian studies of student’s health and thriving (SHoT) also reported increased mental health problems among students [15]. Measured using the Hopkins Symptom Checklist-90 (HSCL-90), 19% of the students (*N* = 13,663) reported serious mental health strain in 2014, which was almost twice the proportion among non-students within the same age group. Women had the largest increase in reporting serious mental health problems, from 16% in 2010 to 25% in 2014, compared to 9 and 12%, respectively, for men. Both studies were cross-sectional.

Many researchers have criticized the findings of decreased mental health and questioned whether this trend is specific for students, and the most well-founded critique came from Hunt and Eisenberg [16]. In a review, they posed the question, “Are mental health problems increasing among college students?” They examined 10 studies in which mental health data from students were compared with findings in the general population and found that both the level and increase in mental health problems in students are similar to those of same–aged non-students. Zivin et al. [17] followed 763 students from 2005 to 2007 and found that the students scored about the same in 2007 as they did 2 years earlier. Approximately 35% were assessed to have a mental health problem. With regard to mental health among persons in professional work, at least an equivalent number of cross-sectional studies have been conducted.

Lelliott et al. [18] suggested that one-sixth of the working-age population suffers from conditions such as depression and anxiety, and another one-sixth suffers from burdens associated with mental health problems, such as worry, sleep problems, and fatigue. In most developed countries, mental illness is now considered the most important cause of absence due to illness, and economic analyses have shown that mental health problems represent large costs to society [19]. In Norway, mental health researchers have estimated that the direct costs of treatment and indirect costs related to early death and retirement from work are roughly 70 billion Norwegian kroner (7 billion Euro) each year [20]. This estimate includes individuals over 16 years of age. In a report from the Norwegian National Institute of Occupational Health (STAMI), empirical mental health data on sub-groups of professionals (health workers, teachers, and social workers) showed that nurses had the highest proportion (21%) of individuals with mental health burden, indicating the need for health care, and teachers came second (11%) [21]. In contrast, a study from our own research group showed a higher mental health burden among teachers (22%) than nurses (15%) 3 years after graduation [22]. However, an important finding was that mental health is better 3 years after graduation, regardless of profession [22–24].

In a review of the evidence-based literature on developing a mentally healthy workplace, Harvey et al. [25] described five general factors that contribute to this. The first, the design of the job, is based in part on Karasek’s job demand and control (JDC) model [26], including demands, control, resources provided, work engagement, and potential for trauma. The second factor is the team/group, including support from colleagues and managers, the quality of interpersonal relationships, effective leadership, and availability of manager training. The third is organizational factors, such as support from the organization, recognizing work, justice, a safe and positive climate in the organization, and the physical environment. The fourth factor is home/work conflict, which is the degree to which conflicting demands from home interfere with work. Finally, the fifth factor consists of individual biopsychosocial factors: genetics, personality, physical and mental health history, and coping style.

While enrolled in university or college, students receive varying degrees of training to manage practical situations in the workplace. However, after graduation, the young professionals meet their responsibilities at work. Very few studies of mental health among students and young professional workers have used longitudinal designs.

The aims of the present study were to investigate change and stability in the levels of psychological distress among healthcare professionals, teachers, and social workers from the end of their study programs until 3 years into their subsequent professional lives and to assess the extent to which psychological distress at the end of the study program, sociodemographic characteristics (age, gender, and civil status), coping with the professional role, the psychosocial workplace environment, and experience of overall quality of life can predict psychological distress 3 years into their professional lives.

## Methods

### Design and data collection

We employed a prospective longitudinal design, examining changes from the end of the students’ study program until 3 years into their professional lives. The data were part of StudData [27] and collected by self-reporting questionnaires from two panels of students (total *n* = 773) in healthcare (*n* = 357, 46.2%), education (*n* = 228, 29.5%), and social work (*n* = 188, 24.3%). The same people were followed as young professionals 3 years later. All 773 participants had valid scores on all variables at both time points. The participants were recruited from six different Norwegian higher education institutions, with the majority (*n* = 434, 56.1%) recruited from Oslo.

### Measures

#### General health questionnaire 12

The GHQ-12 is a widely used self-report instrument for measuring psychological distress and for screening non-psychotic mental disorders [8, 28]. The GHQ-12 has been validated in a large number of studies of the general adult population, clinical populations, and occupational populations, as well as populations of students and young professionals [7, 8, 29–31]. The 12-item version was chosen for the present study and applied as both an independent variable at the end of the study and a dependent variable 3 years after study completion.

Six items on the GHQ-12 are framed positively (e.g., *‘able to enjoy day-to-day activities’*) and six are framed negatively (e.g., ‘*felt constantly under strain*’). For each item, the person is asked to indicate whether he or she has experienced the problem during the last 2 weeks using four response categories: ‘less than usual’, ‘as usual’, ‘more than usual’, or ‘much more than usual’. The GHQ-12 is constructed as a state-measure that is sensitive to changes in mental distress. It is based on a one-dimensional model that assumes that all psychiatric disorders share a common factor. Degree of severity can then be placed on one axis. This one-dimensional model is reflected in the application of a Likert system with scores of 0, 1, 2, or 3. The score range is 0–36, with higher scores indicating more psychological distress and lower scores indicating positive mental health.

A second scoring system, the GHQ-12 case score, is based on a clinical theory assuming that one can identify a clinically meaningful threshold in the dimension of distress as measured by the GHQ-12 [32]. The threshold constitutes the cut-off point at which a clinically significant disorder (case) is reflected in the participant’s score. When using GHQ-12 as a screening instrument, categorical scoring of 0, 0, 1, 1 is employed, resulting in a scoring range of 0–12. Like most GHQ-12 studies that measure mental health problems, we have applied the 4+ threshold. Studies of the validity of the 4+ threshold have been found to have a sensitivity of 84.6, specificity of 89.3, and ROC curve of 0.95 [33]. Goldberg et al. [32] recommended applying the GHQ-12 case scoring system to detect cases in both clinical work and research. The WHO concept of ill mental health, described as the presence of a negative impact on the individual’s quality of life and ability to function adequately, is a more general description of the GHQ-12 case level in principle [5]. We applied both scoring systems.

#### Global quality of life

One item was used, “*How satisfying is your life for the time being*?” The item was scored from 0 (not satisfying at all) to 5 (very much satisfying). This single item has been found to be a valid measure of quality of life in a sample of 5000 therapists [34].

#### Professional role

Orlinsky et al. [34] designed three questions by which to assess a person’s feelings related to his or her professional role (translated from Norwegian to English by the authors): “*How confident are you in your professional role*?” (confidence); “*How good is your theoretical understanding*?” (theoretical understanding); and “*How well do you master the methodical aspects of the work*?” (methodical aspects). All items are scored from 1 (not at all) to 5 (extremely).

#### Job demand, control, and support

Karasek’s JDC model has been theoretically and empirically important for identifying factors contributing to healthy and unhealthy workplaces [25, 26, 35]. Experiencing work with a high demand factor (e.g.*, “My job requires working very fast*”) combined with a low control factor (e.g., “*On my job, I am given a lot of (very little) freedom to decide how I do my work”*) has been shown in many studies to be associated with high psychological distress [36]. The original model has been expanded to include a support factor (JDCS) [37], predicting that jobs with a high support factor (e.g., “*People I work with take a personal interest in me*” and “*People I work with are helpful in getting the job done”*) contribute to decreased psychological distress. We applied the 18-item version of Karasek’s Job Content Questionnaire (JCQ) [37, 38] to measure psychosocial work conditions at the young professionals’ workplaces, including control, demand, and co-worker social support. All of the items of the JCQ have four response categories, and higher scores indicate higher levels of the measured construct.

#### Sociodemographic variables

The three largest professional groups educated in Norwegian universities or university colleges are healthcare workers (including all health education), teachers (including all teaching education), and social workers (including all social work education). Thus, the relevant study programs were merged into larger groups and classified as healthcare, teacher, or social work. The participant’s age in years (continuous variable), gender (female = 1, male = 2), and civil status (not married/no  partner = 1, married/partner = 2) were requested in the questionnaire used at the end of the study program.

### Statistical analysis

All data were entered into the computer program IBM SPSS [39]. Descriptive analyses were performed on all variables using means and standard deviations (SDs), or frequencies and percentages as appropriate. Group differences (between panels and professional groups) were examined with the chi-squared test, independent samples *t*-test, and one-way analysis of variance (ANOVA). In the whole sample and within each of the professional groups, McNemar’s test for categorical variables and paired samples *t*-test were used to identify changes in psychological distress from the end of the study program until 3 years later.

Multivariate linear regression analyses were used to examine individual predictors of psychological distress at the 3-year follow-up. These analyses were performed for all of the professional groups combined and for each of the professional groups separately. The GHQ-12 Likert score at the 3-year follow-up was treated as the dependent variable. Independent variables were entered into the regression model in five steps: 1) psychological distress (GHQ-12 Likert score) at the end of the study program, 2) sociodemographic variables (age, gender, civil status), 3) professional role variables (confidence, theoretical understanding, and methodological aspects), 4) psychosocial workplace environment (demand, control, and support), and 5) global quality of life. Effect sizes (ESs) were calculated by Morris’ [40] formula: σD = σ·2·1-ρ.

Multivariate logistic regression analyses were used to identify factors associated with having psychological distress at case level (i.e., case score ≥ 4). The analyses were performed for all of the professional groups combined and for each of the professional groups separately. The GHQ-12 case score at the 3-year follow-up was used as the outcome (case = 1, non-case = 0). Independent variables were entered in the same order as in the linear regression analyses, but all in one step: psychological distress (GHQ-12 Likert score) at the end of the study program, age, gender, civil status, confidence, theoretical understanding, methodical aspects, demand, control, support, and global quality of life. ESs were calculated as odds ratios (ORs). For all analyses, the level of significance was set at *p* <  0.05.

## Results

At the completion of their study program, the mean age of the students was 24.8 years (SD = 6.5 years), 656 (84.9%) were women, and 518 (67.0%) lived with a spouse or partner. Table 1 shows the proportion of GHQ-12 case scores at the two time points in the total sample and in the professional subgroups. In the total sample, 195 participants (25.2%) belonged to the case group at the end of the study program. The proportion with case-level psychological distress was significantly reduced 3 years later (*n* = 134, 17.3%, *p* <  0.001). Among the healthcare professionals, 94 participants (26.3%) qualified as belonging to the case group at the end of the study program. However, 3 years later the proportion with case-level psychological distress was significantly reduced (*n* = 54, 15.1%, *p* <  0.001). We found the same tendency in the social worker group, in which participants with case-level psychological distress decreased from 49 (26.1%) to 32 (17%, *p* = 0.03) during the 3-year period. The reduction in the proportion of teachers with case-level psychological distress, however, was not significant (*p* = 0.70).

**Table 1:** Proportions of participants with GHQ-12 case scores above the cut-off (GHQ-12 case score ≥ 4) from the end of the study program until 3 years into their professional work lives

Groups	*n*	End of study program	3-year follow-up	Test
		n (%)	n (%)	*McNemar*
Total sample	773	195 (25.2)	134 (17.3)	< 0.001
Healthcare	357	94 (26.3)	54 (15.1)	< 0.001
Teachers	228	52 (22.8)	48 (21.1)	0.70
Social workers	188	49 (26.1)	32 (17.0)	0.03

The changes in GHQ-12 Likert scores for the whole sample and three professional groups are shown in Table 2. In the whole sample, the GHQ-12 Likert scores decreased significantly, though with a small ES, during the 3-year period (*d* = 0.14, *p* <  0.001). In the group-specific analyses, a small yet significant decrease in the GHQ-12 Likert scores was also found for healthcare professionals (*d* = 0.22, *p* <  0.001). The decreases in GHQ-12 Likert scores for the teachers and social workers were not significant.

**Table 2:** Changes in the participants’ psychological distress (GHQ-12 Likert scores) from the end of the study program until 3 years into their professional work lives

Groups	*n*	End of study program	3-year follow-up	Test	ES
		*M* (*SD*)	*M* (*SD*)	*p*	*d*
Total sample	773	11.7 (5.3)	10.8 (4.7)	< 0.001	0.14
Healthcare	357	11.9 (5.4)	10.5 (4.4)	< 0.001	0.22
Teachers	228	11.3 (5.0)	11.1 (5.2)	0.56	0.03
Social workers	188	11.7 (5.2)	10.9 (4.6)	0.08	0.12

Effect sizes (ESs) are calculated by Morris’ (2008) formula: σD = σ·2·1-ρ, see http://www.psychometrica.de/effect_size.html

### Factors associated with psychological distress

The results of the linear regression analyses are given in Table 3. In the total sample, more psychological distress 3 years after study completion was associated with higher psychological distress at the end of the study program (*β* = 0.15, *p* <  0.001), higher levels of job demand (*β* = 0.14, *p* <  0.001), and lower global quality of life (*β* = − 0.46, *p* <  0.001). The full regression model was significant (*F* = 30.4, *p* < 0.001) and explained 30.5% of the variance in psychological distress 3 years into the participants’ professional work lives.

**Table 3:** Factors associated with the participants’ psychological distress (GHQ Likert scores) 3 years into their professional work lives

Independent variables	Total sample (*n* = 773)	Healthcare (*n* = 357)	Teachers (*n* = 228)	Social workers (*n* = 188)
Prior psychological distress				
GHQ Likert score as student	0.15***	0.18***	0.18**	0.08
Explained variance	6.2% ***	8.1% ***	8.9% ***	**1.8%**
Sociodemographics				
Age	0.03	0.10*	−0.05	− 0.01
Gender	−0.06	− 0.08	− 0.05	− 0.09
Civil status	0.02	0.06	−0.03	− 0.01
*R*^2^ change	**0.7%**	**1.8%**	**0.8%**	**1.7%**
Explained variance	6.9% ***	9.9% ***	9.7% ***	**3.5%**
Professional role				
Confidence	0.00	0.19*	−0.04	−0.17
Theoretical understanding	−0.04	− 0.01	−0.16	0.04
Methodical aspects	0.08	0.02	−0.02	0.25*
*R*^2^ change	0.2%	1.2%	0.1%	2.5%
Explained variance	7.1% ***	11.1% ***	9.8% **	6.0%
Psychosocial work environment				
Demand	0.14***	0.12*	0.11	0.18**
Control	−0.02	0.06	−0.14*	0.03
Support	−0.01	−0.18*	0.21*	−0.03
*R*^2^ change	4.6% ***	5.2% ***	6.7% **	5.7% *
Explained variance	11.7% ***	16.3% ***	16.4% ***	11.7% *
Quality of life				
Global quality of life	−0.46 ***	−0.45***	−0.48***	− 0.45 ***
*R*^2^ change	18.8% ***	17.6% ***	19.2% ***	18.5% ***
Explained variance	30.5% ***	33.9% ***	35.6% ***	30.2% ***
Durbin-Watson	1.98	1.96	2.10	1.88

Effect sizes are standardized *β* weights. General Health Questionnaire (GHQ-12 Likert) is scored 0–36 with higher scores indicating more psychological distress; female = 1, male = 2; not married/partner = 1, married/partner = 2 (civil status); professional role variables are scored from 1 (not at all) to 5 (extremely); psychosocial work environment variables are scored as higher scores indicating higher levels of job demand, personal control, and experienced support; global quality of life is scored as higher scores indicating higher quality of life

***p < 0.001, **p < 0.01, *p < 0.05

Among the healthcare professionals, more psychological distress 3 years after study completion was associated with higher psychological distress at the end of the study program (*β* = 0.18, *p* < 0.001), higher age (*β* = 0.10, *p* < 0.05), higher professional role confidence (*β* = 0.19, *p* < 0.05), higher levels of job demand (*β* = 0.12, *p* < 0.05), lower levels of job support (*β* = − 0.18, *p* < 0.05), and lower global quality of life (*β* = − 0.45, *p* < 0.001). The full regression model was significant (*p* < 0.001) and explained 33.9% of the variance in psychological distress 3 years into the healthcare professionals’ work lives.

Among the teachers, more psychological distress 3 years after study completion was associated with higher psychological distress at the end of the study program (*β* = 0.18, *p* < 0.001), lower levels of job control (*β* = − 0.14, *p* < 0.05), higher levels of job support (*β* = 0.21, *p* < 0.05), and lower global quality of life (*β* = − 0.48, *p* < 0.001). The full regression model was significant (*p* < 0.001) and explained 35.6% of the variance in psychological distress 3 years into the teachers’ work lives.

Among the social workers, more psychological distress 3 years after study completion was associated with higher scores on coping with methodical aspects (*β* = 0.25, *p* < 0.05), higher levels of job demand (*β* = 0.18, *p* < 0.01), and lower global quality of life (*β* = − 0.45, *p* < 0.001). The full regression model was significant (*p* < 0.001) and explained 30.2% of the variance in psychological distress 3 years into the social workers’ professional lives. All linear regression analyses had acceptable levels of the Durbin-Watson coefficient.

### Factors associated with GHQ-12 case-level score

The results of the logistic regression analyses are given in Table 4. In the total sample, a higher GHQ-12 Likert score at the end of the study program, experiencing higher levels of job demand, and lower global quality of life increased the risk of having a case-level score indicating psychological distress at the 3-year follow-up. In the healthcare group, a higher GHQ-12 Likert score at the end of the study program, higher age, and lower global quality of life increased the risk of having a case-level score. Among the teachers and social workers, lower global quality of life increased the risk of having a case-level score.

**Table 4:** Factors associated with GHQ-12 case-level psychological distress 3 years into the students’ professional work lives

	Total sample (*n* = 773)		Healthcare (*n* = 357)		Teachers (*n* = 228)		Social workers (*n* = 188)	
	OR	95% CI	OR	95% CI	OR	95% CI	OR	95% CI
Independent variables								
GHQ-12 Likert score as student	1.06**	1.02–1.10	1.12***	1.05–1.19	1.03	0.95–1.11	1.04	0.96–1.14
Age	1.01	0.98–1.05	1.06*	1.00–1.12	0.96	0.90–1.02	1.03	0.97–1.09
Gender	0.72	0.37–1.38	0.36	0.10–1.34	1.20	0.47–3.08	0.21	0.02–1.82
Civil status	0.93	0.59–1.47	1.33	0.61–2.90	0.54	0.24–1.20	1.03	0.40–2.66
Confidence	1.14	0.82–1.57	1.63	0.89–2.96	0.89	0.51–1.57	1.05	0.58–1.88
Theoretical understanding	0.79	0.54–1.14	0.85	0.45–1.60	0.83	0.40–1.70	0.88	0.44–1.75
Methodical aspects	1.23	0.86–1.76	1.33	0.70–2.53	0.75	0.40–1.43	1.43	0.70–2.93
Demand	1.13**	1.04–1.22	1.13	0.99–1.29	1.10	0.92–1.31	1.10	0.94–1.29
Control	1.02	0.95–1.09	1.11	0.99–1.25	0.96	0.84–1.09	1.01	0.87–1.16
Support	0.98	0.94–1.03	0.93	0.85–1.01	1.05	0.97–1.14	0.94	0.87–1.03
Global quality of life	0.34***	0.27–0.44	0.29***	0.20–0.43	0.24***	0.14–0.41	0.42***	0.27–0.65
Adjusted model parameters								
Model χ^2^	150.62***		87.81***		57.88***		34.69***	
Nagelkerke *R*^2^	0.29		0.38		0.35		0.28	
Cox & Snell *R*^2^	0.18		0.22		0.22		0.17	
Hosmer-Lemeshow χ^2^	7.71		7.22		6.35		6.40	

Effect sizes are standardized *β* weights. General Health Likert Questionnaire (GHQ-12) is scored 0–36, with higher scores indicating more psychological distress; female = 1, male = 2; not married/partner = 1, married/partner = 2 (civil status); professional role variables are scored from 1 (not at all) to 5 (extremely); psychosocial work environment variables are scored as higher scores indicating higher levels of job demand, personal control, and experienced support; global quality of life is scored as higher scores indicating higher quality of life

***p < 0.001, **p < 0.01, *p < 0.05

## Discussion

The main result of this longitudinal study was that psychological distress decreased from the end of the study programs until 3 years into the participants’ subsequent professional lives. Thus, our findings indirectly support the assumptions about higher levels of mental problems among students. Factors important for reduced psychological distress differed between the groups, but one factor, the current experience of quality of life, contributed to lower psychological distress with a moderate to large ES in all analyses.

The findings in this study are in line with previous studies showing that the transition from study to work is associated with better mental health in most student groups, independent of profession and gender [22, 24]. They are also in line with Harvey et al.’s review of the evidence-based literature suggesting mentally healthy workplaces [25]. However, we were interested in gaining a better understanding of the known tendency for reduced psychological distress from study to work. Therefore, we examined the three different groups with different factors associated with mental health 3 years into their professional lives. One factor of importance was the level of psychological distress when finishing the study. This had a significant impact on subsequent psychological distress among the healthcare professionals and teachers, but not among the social workers. However, the variance explained by the GHQ-12 Likert score as a student was modest, indicating that this factor alone is insufficient for explaining subsequent psychological distress.

Demand, control, and support are all factors defined as key work characteristics associated with both positive and negative outcomes [41]. Positive outcomes include motivation and learning, whereas negative outcomes include illness and strain, such as psychological distress. In a work context, demand can be understood as psychological, physical, cognitive and organizational constraints, work load, work environment, and pressure, not least of which is time pressure [26, 42]. Individuals who experience excessive job demands may feel like losing their personal resources and the capacity to cope with the demands. Demands may be stressful due to a feeling of not having the time or ability to do the tasks as expected. On the other hand, job control is one’s own control over tasks and is defined as the opportunity for decision authority or autonomy in work [41]. According to Bakker and Demerouti [43], job control can be a resource that allows the individual to deal with the work demands. Social support is an interaction between the employee and his or her supervisor and co-workers and is valuable according to task assistance, access to information, and social companionship. This is also called the employee’s social capital [41]. Such support may be experienced as a job resource [43].

In our study sample, higher levels of job demand had a significant impact on psychological distress. When dividing the sample into the three groups, demand was associated with a higher level of psychological distress among the participants in the healthcare and social work groups. An explanation for this may be that employees in health care and social work have a heavy workload related to their clients’ mental and physical health and well-being. In addition, the time they have available for each patient or client is limited. It is reasonable to assume that the association between job demand and higher psychological distress in these two groups may be due to an experience with the potentially detrimental consequences of a high workload and time pressure in these professional fields. In anticipation of their potentially harmful consequences for clients, high job demands may give rise to feelings of ineptness, reduced coping, and higher distress levels.

Such thinking is in line with Lazarus and Folkman [44], who demonstrated that perceived coping resources contribute to the individual’s stressor perception. Previous studies underscore that workplace demands and experiencing a loss of resources may produce psychological distress. In turn, such distress may reduce the ability to meet the demands and result in loss of energy and reduced health [43, 45, 46]. Although there may be high levels of job demand in a classroom when working with children and adolescents, in addition to all preparations and follow-ups, an explanation for why demand did not significantly impact psychological distress in the teacher group is needed. As previously noted, the consequences of not meeting the demands in every situation may not be as severe as when working with vulnerable clients. Compared to the health care professionals, teachers’ ‘clients’ are primarily healthy children, whereas the health care group is confronted with life and death. In addition, the workload may be experienced differently by the young teachers compared to their counterparts in healthcare and social work.

Only in the teacher group, higher levels of control were significantly associated with reduced psychological distress. As described above, job control is characterized by the experience of having control over tasks, as well as an opportunity to exercise decision authority and autonomy in the work. Therefore, the results may indicate that, for the teachers, greater opportunities to think of alternative solutions and the ability to make spontaneous decisions and use different pedagogy are important for their distress levels. As such, job control can be experienced as a resource that allows the teacher to deal with the demands related to working as a teacher.

In the health care group, support was associated with better psychological health, whereas the association was the opposite in the teacher group. In health care, there is a tradition that seniors supervise and support young colleagues, regardless of how and when the demands are (too) heavy. Well-functioning systematic support may prevent the development of psychological ill health and generally contribute to higher levels of social capital. In addition, more confidence, as part of the professional role, was significantly associated with better mental health among healthcare workers. Regular supervision, being part of a hierarchical system with senior colleagues, and often working together with co-workers may contribute to explaining these results. In addition, both the health care professions and social worker traditions normally apply supervision both during education and in the first years of professional work. Klette and Smeby [47] and Scheerens [48] have reported in their research on teachers that collegial feedback for teachers is rare. It may be that the pattern of support in teaching is less systematic and less targeted to solving challenges in the workplace and more tailored towards individuals with expressed needs at the personal level. If this were the case, more support would be reported by those experiencing higher levels of distress.

Compared to the other two groups, the teachers exhibited a smaller reduction of psychological distress from the end of their study to 3 years after starting as a young employee. However, a significant difference was only found for the healthcare group. The reasons for these differences may be related to the above arguments according to job demand, control, and support.

Better mental health as measured by the GHQ-12 was associated with experiencing a higher quality of life in all three groups. This finding seems to be in line with the theoretical expectation that good mental health as measured by the GHQ-12 is strongly associated with good quality of life, and vice versa. For example, Næss et al. [49] defined quality of life as mental well-being based on the person’s cognitive and affective experiences and if these are positive or negative. In principle, GHQ 12 measures both positive and negative mental health.

Næss et al. [49] described global quality of life to include an individual’s satisfaction, happiness, meaning, and realization of goals in their own lives, and it is the individual’s subjective opinion that is requested. According to Næss et al. [49], it is the individual’s own opinion about his or her life that is important. She emphasized that mental well-being is related to happiness, whereas satisfaction is associated with the individual’s personal appraisals. Her definition includes both cognitive and affective aspects, including thoughts, appraisals, feelings, and emotions. Being satisfied with life as a whole seems to cause good mental health. On the other hand, it may be that good mental health improves the quality of life and experience of having a good life. In general, demographic variables had a small impact on psychological distress. This finding is line with previous research among young professional workers [22, 24].

## Study strengths and limitations

A strength of this research is the longitudinal design and transition between the end of a study program to 3 years into professional life. Another strength is the use of two scoring principles: case and Likert score. The sample size, as well as participants being from six different universities and colleges from different parts of Norway, are also strengths. Furthermore, the sample size provided an opportunity to investigate associations with psychological distress/mental health while controlling for several variables. However, the predictors or independent variables were only measured at 3 years and may be seen as a limitation because we cannot decide cause and effect, only associations. Another limitation may be that the overall quality of life is measured with one item.

## Conclusion and implications

The main findings were that psychological distress was reduced from the end of the study program to 3 years into professional work in the health care and social work groups on the case level, but not among the teachers. A strong association was found between overall quality of life and mental health in the total sample and all three groups, but the other independent variables were differentially associated with psychological distress at 3 years in the different groups. Psychological distress at the end of the study program and psychosocial work environment were the most important variables.

## Data Availability

The data supporting the findings of this study are available from Oslo Metropolitan University, but restrictions apply to the availability of these data, which were used under license for the current study and are not publicly available.

## Abbreviations

ACHA: American College Health Association
ANOVA: Analysis of variance
BDI: Beck Depression Inventory
GHQ-12: General Health Questionnaire 12
HSCL-90: Hopkins Symptom Checklist-90
JCQ: Karasek’s Job Content Questionnaire
JDC: Karasek’s job demand and control
OR: Odds ratio
SD: Standard deviation
SPSS: Statistical Package for the Social Sciences
STAMI: Norwegian National Institute of Occupational Health
StudData: Database for Studies of Recruitment and Qualification in the Professions
WHO: World Health Organization
